# Do no harm? How health care management research can advance environmentally sustainable health care

**DOI:** 10.1097/HMR.0000000000000503

**Published:** 2026-08-06

**Authors:** Maike V. Tietschert, Jülide N. Çelik, Alexander Negron Price, Sara J. Singer, Andrew N. Garman, Erik M. Van Raaij

**Affiliations:** Maike V. Tietschert, PhD, Assistant Professor of Sustainable Health Systems, Erasmus University Rotterdam, Erasmus School of Health Policy & Management, Rotterdam, The Netherlands; Jülide N. Çelik, MSc, PhD Student, Erasmus University Rotterdam, Erasmus School of Health Policy & Management, Rotterdam, The Netherlands; Alexander Negron Price, MS, Sustainability Research Assistant and Medical Student, Rush University, Department of Health Systems Management, Chicago, IL; Sara J. Singer, PhD, MBA, Professor of Health Policy, Stanford University School of Medicine, Stanford, CA; Andrew N. Garman, PSYD, MS, Professor of Health Systems Management, Rush University, Department of Health Systems Management, Chicago, IL; Erik M. Van Raaij, PhD, Professor of Sustainable Procurement in Health Care, Erasmus University Rotterdam, Erasmus School of Health Policy & Management, Rotterdam School of Management, Technology & Operations Management, Rotterdam, The Netherlands

**Keywords:** health care management research, research framework, sustainability, sustainable health care

## Abstract

**Issue:**

Health care organizations face pressure to advance environmental sustainability. Core challenges include pursuing distant-future goals (e.g., climate neutrality by 2050), leading sustainability initiatives beyond formal authority, and making mitigation decisions whose benefits accrue in the future and outside the organization.

**Critical Theoretical Analysis:**

Dominant management theories tend to treat the future as a strategic context or planning horizon rather than as an object of sustained organizational work. Long-term orientation is often assumed rather than explained. Leadership is conceptualized as hierarchical or professionally anchored, leaving distributed and extra-professional leadership weakly theorized. Moreover, prevailing theories assume alignment between decision-makers and beneficiaries, privileging value creation and capture while inadequately addressing harm reduction and externalized and future-oriented environmental benefits and costs.

**Insight/Advance:**

We suggest a shift from treating the future as a given condition to be anticipated or adapted to, toward actively pursuing distant futures as ongoing organizational work. We conceptualize sustainability leadership as distributed across fragmented authority structures and reframe environmental sustainability as a governance challenge of managing asymmetric costs and benefits alongside clinical priorities. Reframing of sustainability leadership provides opportunities for healthcare management research to advance environmentally sustainable health care through managerial work.

**Practice Implications:**

We highlight the need for health care managers to actively sustain future-oriented goals over time, lead sustainability initiatives without relying solely on formal authority, and develop decision and performance frameworks capable of integrating temporally and spatially uneven payoffs.

## Issue

Health care systems globally are grappling with a profound paradox: while health care professionals’ primary task is to promote health and adhere to the oath of “*do no harm*,” health care operations contribute to environmental degradation, threatening the health of current and future generations (Eckelman et al., 2020). Health care systems contribute an estimated 4%–5% of global greenhouse gas emissions (MacNeill et al., 2020), placing health care among the highest emitting sectors worldwide. In the United States, the sector is responsible for ~8.5% of total US emissions, with health impacts of a comparable magnitude to medical errors and other leading causes of death (Eckelman et al., 2020). US health care facilities produce an estimated 33 pounds of waste per bed per day (Jain & LaBeaud, 2022), along with high levels of water, land and raw material use that rival the most resource-intensive sectors.

In response, national and international frameworks encourage health care organizations and systems to commit to ambitious environmental goals, often set in the distant future. Examples include climate neutrality by 2050 and circular models of care delivery (e.g., European Union’s Net-Zero Industry Act, U.S. Health sector climate pledge, Inflation Reduction Act of 2022), which promote use of sustainable and renewable materials and reuse or recycling of products and raw materials to minimize resource input, waste, emissions and energy leakage by slowing, narrowing, and closing material and energy loops (Bocken et al., 2016). Slowing refers to extending the life span of products or materials, for example, through reuse, repair or refurbishing. Narrowing means using fewer materials and less energy in the first place, for example, through smarter design. Closing means recovering materials and energy and cycling it back into production at the end of use, for example, through recycling, so that nothing or as little as possible is wasted.

These goals, recently coined distant-future goals (Feuls et al., 2025), are inherently forward-looking: they extend far beyond typical planning cycles, managerial tenures, and accountability horizons. Yet, environmentally unsustainable practices persist because responsibility for addressing them is fragmented across several nested levels of the system, like other challenges in health care (e.g., Goodrick & Reay, 2016). At the clinical process level, the immediate concern rests primarily with clinicians and clinical leaders, who choose among technologies, products, and services and, in doing so, influence the environmental impact of the care they provide. At the organizational level, executive teams and governing boards determine infrastructure investments and procurement policies that directly shape a hospital’s environmental footprint. At the interorganizational level, global supplier networks determine product design and manufacturing impact and, in turn, the clinical choices clinicians can make. Finally, at the field level, reimbursement and regulatory systems prioritize acute patient and cost outcomes, while leaving environmental harms unrepresented (Eckelman et al., 2020).

This multi-level structure means that the transition toward environmental sustainability is not the job of any single profession or organization. Rather, it requires coordinated action among policymakers, suppliers, clinicians, administrators, and patients. Within this constellation of actors, however, healthcare managers occupy a distinctive position of accountability. They are formally charged with stewarding organizational resources, safeguarding long-term institutional viability, and integrating diverse performance objectives (Mintzberg, 1971). Managers’ control over financial resources gives them leverage to influence supplier behaviors, including the adoption of environmentally sustainable strategies (Lavoie et al., 2024). Consequently, they are the actors expected to convert diffuse sustainability ambitions into concrete operational programs (Beusch et al., 2022). Importantly, in health care, these responsibilities extend beyond the walls of the healthcare organization: managers must work through complex supplier networks and contracting systems to reduce the environmental impact of the products and services their organizations purchase and provide (Lenzen et al., 2020; MacNeill et al., 2020).

Thus, while advancing sustainable health care practices ultimately depends on the interaction of many stakeholders, health care managers are pivotal integrators of the transition. They are expected to align environmental goals with clinical priorities and economic constraints and to justify investments whose benefits accrue in the distant future or outside traditional financial accounts. Yet, lack of top-level management support is frequently reported as a barrier, slowing down progress toward environmental mitigation (van Schie, 2024), here defined as human interventions to reduce environmental impact.

We argue that, despite emerging managerial responsibility for environmental sustainability in health care management research, it has not yet fully theorized the type of managerial work required to advance environmental sustainability, particularly mitigation, due to three issues. We suggest that mitigation is particularly challenging because mitigation requires health care organizations to take actions not typically within the jurisdiction of clinical care, for which people working in clinical environments are not typically trained, and for activities not considered core to the organizational mission.

### Issue 1

Managing for environmental sustainability in any organization through mitigation practices, health care being no exception, requires organizations to actively pursue distant-future goals and to sustain their relevance over extended periods (Feuls et al., 2025). While management scholarship frequently invokes long-term orientation, the future is often treated as a given that requires anticipation and control, for example, in the field of strategy. However, recent work on organizations’ orientation toward the future suggests that *distant futures* are difficult to maintain as actionable reference points in everyday decision-making (Hernes & Schultz, 2020). Management research has only begun to examine how organizations construct and mobilize such futures (Feuls et al., 2025), and it has rarely explored how these processes shape concrete outcomes. In health care, where environmental sustainability goals extend decades into the future and intersect directly with population health, this theoretical gap becomes particularly consequential.

### Issue 2

Advancing environmentally sustainable health care depends on forms of leadership outside traditional managerial authority and clinical expertise. Health care management theory has historically located decision-making authority either in hierarchical managerial structures, as emphasized in New Public Management (Simonet, 2015), or in professional expertise, in particular that of clinicians, as highlighted in institutional and professional governance perspectives (Freidson, 1988). Institutional theory, for example, shows how healthcare organizations are shaped by multiple and sometimes competing logics (Goodrick & Reay, 2016), including market-oriented and professional logics. However, the market logic that often guides managerial attention may prioritize efficiency and cost containment over the upfront investments required to advance circular models of care delivery. At the same time, professional logics may focus primarily on the immediate health effects of care delivery decisions for current patients, while giving less attention to the long-term, distributed consequences of environmentally unsustainable practices.

Theory on hybrid organizing advances this debate by recognizing the coexistence of multiple logics—organizing principles associated with different sectors that legitimize behavior (Thornton & Ocasio, 2008) and shared authority within organizations (Battilana & Dorado, 2010; Denis et al., 2015; Pache & Santos, 2013). However, environmental sustainability introduces knowledge domains, such as carbon accounting, life-cycle analysis, and sustainable procurement, that fall outside established jurisdictions in health care (Eckelman & Sherman, 2016).

### Issue 3

Managing for environmental sustainability requires decision-making under conditions of geographically and temporally asymmetric costs and benefits (Slawinski & Bansal, 2015). Mitigation efforts often impose immediate and localized costs while generating benefits that are diffuse, delayed, and external to the organization (Hahn et al., 2015). Current management theory highlights tensions among competing logics but does not adequately address how managers should govern decisions when benefits are spatially and temporally displaced from those bearing the costs (Hahn et al., 2015). For example, stewardship theory suggests that managers can act as custodians of long-term organizational and societal value, prioritizing collective welfare over short-term self-interest (Davis et al., 1997; Hernandez, 2012). Rather than assuming opportunistic behavior, stewardship theory positions managers as intrinsically motivated to protect resources on behalf of future beneficiaries, even when those beneficiaries are geographically or temporally remote. Stakeholder theory, meanwhile, broadens the managerial obligation beyond shareholders to encompass all parties affected by decisions (Freeman, 1984), providing a framework for identifying whose interests must be weighed when costs and benefits fall unevenly across groups. Together, these perspectives suggest that responsible governance requires both the dispositional orientation to act as a steward and the relational mapping of who bears risk across space and time. However, existing theories offer limited guidance when prosocial obligations themselves are at risk of being in conflict, situations where the interests of proximate and future beneficiaries could compete. Such tensions do not reflect failures of managerial motivation or stakeholder identification, but represent genuine moral trade-offs between competing goods, where prioritizing one legitimate claim can compromise another. Hence, existing perspectives provide limited guidance for managerial decision-making under conditions of misaligned responsibility, authority and benefit realization.

Taken together, these limitations indicate that prevailing health care management theories may be insufficiently equipped to guide managerial action toward environmental sustainability in health care. While they illuminate important structural tensions, they insufficiently account for the long temporal horizons of environmental goals, the distributed and extra-professional nature of sustainability expertise, and the asymmetric distribution of costs and benefits that characterize sustainability transitions in health care. Addressing these challenges requires theoretical approaches that more explicitly engage with temporality, dispersed knowledge, and decision-making under conditions of fragmented accountability.

The purpose of this paper is to (1) critically analyze why management theories fail to equip healthcare managers for environmental mitigation and (2) articulate theoretical advances that health care management research can offer to advance the transition toward sustainable health care systems. Our analysis is inspired by insights derived during a Professional Development Workshop (PDW) offered by the Health Care Management Division of the Academy of Management at the organization’s 2024 Annual Meeting, in Chicago, Illinois, USA. The PDW focused on developing a framework and agenda for health care management research to advance environmentally sustainable health care practices. The PDW gathered 41 health care management scholars and practitioners, with varying degrees of experience and expertise in environmental sustainability. Most participants (n=29) worked for US-based research institutions. Other countries represented included Canada, Germany, Italy, Malaysia, Sweden, the Netherlands, the United Kingdom, and the United Arab Emirates. When participants registered, they provided their contact details, affiliation, role, and listed theories, methods and types of data they use in their research. Based on similar theoretical interests, participants joined one of six groups broadly related to implementation science, institutional theory, organizational learning, the resource-based view of the firm, organizational behavior, and organizational theory. We informed participants about the intent to synthesize the findings of the workshop in a publication. Each roundtable, led by a PDW organizer, used an A0-format worksheet to structure brainstorming on “The Opportunity for Healthcare Management.” Participants formulated research questions on sustainable health care systems, identified relevant theories, and listed suitable methods and data types following a mind-mapping approach. A representative from each table then presented their findings.

After the PDW, we transferred all notes to MS Excel. The first and last author categorized the theories, methods and research questions based on broader research fields within the health care management literature. Participants mostly discussed research questions and theoretical approaches. Methods or data sources were only discussed for some areas. Hence, we limited our synthesis to relevant research questions and theories. We grouped similar ideas together and added information based on our understanding of the field and literature. All authors met to derive a final set of research areas and questions. While this produced a rich set of questions to be addressed, which can be requested from the authors, this paper discusses those issues that are distinct in the transition to environmentally sustainable health care systems, and that are not well addressed by existing health care management research theories and methods.

## Theoretical Analysis

Management scholarship outside the healthcare sector has engaged environmental sustainability for more than three decades (cf. Etzion, 2007), particularly within strategic management, organization theory, and corporate responsibility research (e.g., Orlitzky et al., 2011). Taken together, this body of work has significantly advanced the understanding of why and when organizations adopt environmental practices, yet theoretical and conceptual gaps remain.

### Under-theorization of Distant-future Pursuits

Environmental sustainability, especially mitigation, requires organizations to engage with distant-future goals whose realization depends on sustained action over decades. Management theory has long acknowledged the importance of the future through concepts such as strategy, planning, foresight, and long-term orientation. At first glance, this might suggest that the temporal challenge of sustainability is already well understood.

However, most management theories treat the future as a background condition rather than an object of sustained organizational work. Classic strategy research conceptualized management as purposeful action toward anticipated future states, supported by planning and control systems (Ansoff, 1965). Later, the resource-based view (Barney, 1991) and the dynamic capabilities framework (Teece et al., 1997) conceptualize the future primarily as a space of uncertainty and opportunity. Long-term investments are justified insofar as they are expected to generate future organizational benefits, such as competitive advantage, resilience, or superior performance (Flammer & Bansal, 2017). Even work that explicitly critiques short-termism tends to frame long-term orientation as a strategic posture rather than as an ongoing organizational accomplishment (Bansal & DesJardine, 2014; Slawinski & Bansal, 2015). These perspectives help explain incremental investments that resemble other long-term investments, such as energy-efficient buildings, waste reduction programs, or process innovations with calculable payoffs.

Institutional and stakeholder theories similarly privilege present-oriented temporalities. Stakeholders are defined by their current salience, power, and legitimacy, while institutional legitimacy is assessed relative to prevailing norms and expectations (DiMaggio & Powell, 1983; Suchman, 1995). Future beneficiaries, central to environmental mitigation, rarely enter these frameworks.

The performance measurement literature illustrates both the promise and limits of dominant temporal assumptions. Central to performance measurement is the idea that organizations can use indicators to craft narratives about the future and stabilize organizational identity. Basically, futures can be kept salient through calculative devices. However, climate-neutrality targets to 2050 are not simply quantitatively uncertain (e.g., how many tons of CO_2_ must be reduced or removed) but also qualitatively uncertain (Feuls et al., 2025): it is unclear which technologies, care models, or supplier arrangements will eventually constitute a sustainable future.

Finally, the distant-future goal literature explains individual persistence. Bateman and Barry (2012), for example, show that people can sustain motivation to pursue goals that extend over decades through identity anchoring and self-regulation. Issue selling research shows how individuals mobilize organizational attention toward prosocial issues through relational and rhetorical strategies (Dutton & Ashford, 1993; Wickert & De Bakker, 2018). Similarly, Meyerson, 2003 work on tempered radicals explains how motivated actors can advance change from within institutional constraints. This work is relevant because mitigation in health care will also require sustained action by diverse actors (e.g., clinicians, suppliers, regulators, patients). It helps explain the successes of green teams, sustainability champions, and engaged leaders in advancing environmental initiatives in health care. Yet while there may also be implications for teams and organizations (Bateman & Barry, 2012), these frameworks tend to be built around individual agency and do not explain how managers can pursue sustainability in the nested nature of health care, where sustainable practices must be enacted across interdependent levels, from clinical practice and procurement to organizational strategy, supply chains, and policy environments (Eckelman et al., 2020). In this context, sustainability is likely not reducible to individual agency; it requires coordinated change across distributed actors within and beyond organizational boundaries, including suppliers, regulators, and policymakers.

In fact, recent work on organizational futurity helps clarify why current approaches fall short. Schultz and Hernes (2013) argue that organizations are constituted through ongoing constructions of the past and the future; strategic change depends on how actors keep particular futures salient in the present. Feuls et al. (2025) show that futures are not simply anticipated but actively constructed, maintained, and negotiated in the present. Importantly, their research demonstrates that organizations tend to privilege near futures that can be operationalized within existing temporal structures, such as planning cycles, budgets, and performance metrics, while distant-future goals are more fragile and prone to symbolic treatment.

Hence, the challenge of sustainability is not merely that organizations lack long-term goals, but that management theory and health care management theory have not sufficiently explained how distant futures are kept relevant, actionable, and influential over time. With some notable exceptions (e.g., Feuls et al., 2025), much sustainability research assumes the presence of future goals (e.g., climate neutrality by 2050) without examining the organizational work required to sustain them across leadership changes, competing priorities, and shifting institutional environments.

### Extra-professionalism and Distributed Leadership

A second limitation of dominant management theories concerns their treatment of leadership, particularly in contexts where the core object of change is extra-professional, that is, outside the jurisdiction of a professional group. Environmental sustainability in health care depends on coordinated action across organizational, professional, and institutional boundaries and requires expertise that lies outside the traditional jurisdiction of clinical and managerial professions. Key sources of environmental impact, including clinical practice, procurement, infrastructure, and regulation, are governed by actors who do not fall neatly under managerial authority.

Despite this reality, most management theories implicitly assume that organizational change occurs within domains where the relevant expertise, authority, and legitimacy are already institutionally established (Reay & Hinings, 2009). Strategy and change theories presume that managers can mobilize resources and direct action with recognized organizational mandates (Eisenhardt & Martin, 2017; Teece et al., 1997). Leadership theories similarly emphasize influence exercised by actors whose authority is grounded in formal position or recognized expertise.

Health care management scholarship has been profoundly shaped by institutional theory. Institutional theory acknowledges professional dominance in sectors such as health care, highlighting how clinical logics constrain managerial discretion (Freidson, 1988; Reay et al., 2017) and how new practices must be legitimized within existing fields (Greenwood et al., 2011). Collectively, this body of literature is informative in understanding how health care managers can advance change by fitting new concerns into existing systems and by mobilizing coalitions among actors with different forms of legitimacy. It also provides useful strategies for how organizations can deal with competing logics. For example, Goodrick and Reay (2016) outline a continuum of organizational strategies for managing institutional complexity. These range from full segmentation of logics (compartmentalizing responses in separate units or routines) to full integration (hybridizing disparate logics into a single decision system), to partial integration. Short of full integration, the authors identify three mechanisms through which organizations can productively combine logics: reinterpreting practices to render them compatible with other logics, leveraging existing synergies between conflicting principles, and enabling frontline innovation that blends logics in day-to-day work. They caution that both extremes are difficult to achieve, just as it would be unrealistic to make clinical decisions based solely on sustainability criteria without regard to patient safety or cost, it is equally unlikely that organizations can fully insulate sustainability work from established procurement templates. The implication is that partial integration provides the most feasible pathway for managers to embed environmental aims. In practice, sustainability initiatives can be narrated in ways that resonate with prevailing logics, for instance, as strategies to prevent harm to population health (professional logic), as programs to reduce waste and energy expenditure (market logic), or as critical reviews of technologies whose clinical benefits are insufficient to justify their environmental intensity (professional logic). Such reinterpretations expand zones of legitimacy for healthcare managers while avoiding the untenable requirement that any single logic dominates decision-making. However, this literature typically assumes that contested issues fall within professional domains (e.g., quality, safety, evidence-based practices). Environmental sustainability challenges this assumption.

Environmental mitigation in health care is extra-professional in at least three ways. First, it lies outside the historically established jurisdiction of clinical professions, whose expertise is oriented toward diagnosing and treating individual patients, not managing environmental externalities. Second, it is only weakly embedded in professional training, standards, and licensure, leaving clinicians without clear professional scripts, competencies, or mandates for action. Third, environmental sustainability is commonly framed as peripheral to “core care”, rendering it vulnerable to marginalization when professional priorities are invoked—not necessarily because it contradicts professional values, but because it lacks professional anchoring.

Importantly, the extra-professional status of sustainability does not automatically imply a misalignment with professional commitments to protect health. Rather, it reflects the absence of jurisdictional authority and accountability infrastructures through which such commitments are enacted. Existing frameworks for combining different institutional logics can help expand legitimacy, for example, framing waste reduction as cost efficiency, but also carry risks. Environmental projects may disrupt established clinical routines and shift responsibility to individual professionals, potentially increasing workload and perceived uncertainty about safety. Because sustainability is currently viewed as extra-professional and oriented toward distant goals, institutional theory, at least in health care management, can predict these tensions, but less so how to redesign internal roles so that leadership and tasks are fairly and effectively distributed.

As a result, health care managers face a leadership challenge that is not well captured by existing theories: they must advance sustainability in a domain where neither managerial authority nor clinical expertise provides clear legitimacy. Managers cannot rely on hierarchical control, but they also cannot defer to professional authority, because sustainability goals are not owned by any single profession in health care.

Dominant management theories are insufficiently equipped to address this condition. Strategy and change models assume that resistance reflects misalignment or insufficient incentives, rather than the absence of recognized professional jurisdiction. Institutional theory explains why professional logics resist external intrusion, but it offers limited guidance on how new, extra-professional concerns become integrated into existing professional and organizational arrangements. At the level of everyday work, sustainability is therefore often experienced as extra-role, as additional tasks, voluntary initiatives, or peripheral responsibilities. Analytically, however, the more consequential issue is that sustainability is extra-professional: weakly embedded in professional jurisdictions, training, standards, and accountability systems. Focusing on extra-professionalism avoids attributing responsibility to individual clinicians and instead foregrounds the structural conditions under which sustainability remains optional, individualized, and institutionally fragile.

Consequently, leading for sustainability in health care requires forms of leadership that extend beyond both managerial control and professional expertise. It involves boundary-spanning work that creates new zones of legitimacy, where environmental considerations can be discussed, negotiated, and acted upon without being dismissed as irrelevant to professional identity or mandate. While the management literature has begun to acknowledge distributed leadership, it has rarely examined leadership in extra-professional domains, where authority is fragmented, and expertise is contested or emergent.

### Decision-making Under Asymmetry

A third theoretical limitation concerns the rationality assumptions embedded in dominant management theories. Environmental sustainability in health care presents a distinctive decision-making problem because the costs and benefits of climate mitigation are unevenly distributed across time, space, and organizational boundaries. Most management theories implicitly assume that decision-makers evaluate alternatives based on outcomes that are observable, attributable, and captured within organizational performance systems. Economic theories, such as contract theory (Hart & Holmström, 1987) or agency theory (Eisenhardt, 1989), emphasize incentive alignment through pricing and contracts; corporate social responsibility research (Aguinis & Glavas, 2012; Wagner et al., 2009) assumes that organizations can internalize social value; public value theory (Moore, 1997), often applied to nonprofit organizations, is focused on the organization’s role in creating public value within their authorized environment; stakeholder theories (Freeman, 1984) presume that affected parties can exert influence over decisions (Bridoux & Stoelhorst, 2022); and operations management, such as scientific management (Taylor, 1911) or lean six sigma (Womack and Jones, 1990), focuses on efficiency gains that reduce resource use. Across these perspectives, decision-making is modeled as a process in which relevant costs and benefits (whether in economic or social terms) are sufficiently represented to allow rational trade-offs.

Environmental harms associated with health care, however, such as greenhouse gas emissions, waste streams, and resource depletion, are largely externalized, while many benefits of mitigation accrue in the distant future or outside the organization altogether (Eckelman & Sherman, 2016). Industrial and environmental economics help conceptualize this asymmetry by modelling externalities, path dependence and lock-in effects that arise from long-lived infrastructure and technology choices (Unruh, 2000). Industrial ecology further extends the decision frame by tracing life-cycle impacts embedded in products and services (Graedel & Allenby, 2010), showing how decisions taken at the point of care import upstream environmental burden. These perspectives clarify why sustainability-relevant costs and benefits are systematically misaligned with hospital accounting systems, but they offer limited guidance on how managers should decide when such impacts remain unpriced and uncertain.

The asymmetry becomes particularly consequential because current health care decisions generate distributed effects beyond the organization. Health care management research offers rich insights into interorganizational relations, such as indicated by the rich body of research on social networks (cf. Peeters et al., 2023). Related work emphasizes that managerial decision-making is embedded in established interorganizational and intraorganizational relationships. These perspectives explain how managers can mobilize coalitions, coordinate action across departments, and intervene strategically within the fields to which hospitals are connected. Yet the limitation of these theories mirrors that of institutional accounts: because networks are conceptualized and analyzed based on existing, formal relationships, they privilege stakeholders who already transact with or evaluate hospitals. They do not allow for simulating the interests of future or voiceless beneficiaries, including communities in already disadvantaged regions of the world that bear the health consequences of climate change without any direct relationship to other actors.

Emissions and waste generated by resource-intensive care in health systems of the global north disproportionately burden regions already facing social, economic, and environmental disadvantages. Environmental harms are displaced geographically through global production and disposal systems and the nature of climate change, and temporally through impacts on future populations with no representation in present decision-making processes (Bianco et al., 2024). From decision-making perspectives, this means that many of those who bear the greatest health consequences of unsustainable care are absent from the evaluation frameworks that guide managerial choices. This puts to the test even prosocial frameworks such as stewardship theory. Stewardship theory assumes that leaders are motivated by long-term orientation and a shared sense of responsibility toward multiple stakeholders, expressed through pro-social behavior (Davis et al., 1997; Hernandez, 2012). Yet in health care, this assumption encounters a structural tension: care delivery itself constitutes a form of prosocial behavior, and the steward’s relational and ideological obligations are concentrated on beneficiaries of current services, that is, patients that health care organizations serve. Current patients are visible, proximate, and bound to clinicians and managers through both professional duty and relational currency. Yet this concentration of obligation leaves little structural room for those who are absent, meaning future generations and geographically distant populations who bear disproportionate environmental burdens, but who lack the proximity, voice, or institutional standing that would naturally bring them within the steward’s field of moral attention. Institutional theory supports this: beneficiaries who lack voice or institutional standing are unlikely to influence organizational priorities, while dominant professional and efficiency logics continue to structure what counts as a good decision (Reay & Hinings, 2009).

Research on performance measurement further underscores how difficult it is to correct such asymmetries. Performance metrics do not merely report outcomes; they shape organizational attention and identity by defining which benefits are visible and legitimate (Grimes, 2010). Health care organizations are characterized by strong identities centered on immediate patient and population health, safety and cost control. While sustainability indicators may introduce new evaluation dimensions, these compete with entrenched measures that govern investment decisions, technology adoption, and clinical prioritization. As a result, managers face a persistent dilemma: mitigation efforts may appear inefficient or risky within prevailing decision frameworks even when they are justified from a societal or long-term health perspective.

Currently, health care management theory provides limited guidance on how to weigh present clinical benefits against distant environmental harms, how to justify investments whose returns lie outside organizational boundaries, or how to sustain mitigation efforts when evaluation systems do not include related outcomes. For example, while frameworks such as Comparative Effectiveness Analysis consider future benefits, they are often discounted compared with current benefits (Meltzer et al., 2010) and still focused on near futures rather than distant ones. Addressing these gaps requires a decision-centered framework that explicitly theorizes asymmetric costs and benefits as a defining feature of sustainable health care management, rather than as a temporary misalignment to be corrected through existing tools.

Taken together, this analysis suggests that the difficulty of advancing environmental sustainability in health care is not primarily a problem of sectoral complexity or managerial resistance. Rather, it reflects unresolved limitations in how management theory conceptualizes distant futures, leadership without authority, and decision-making under asymmetric value conditions across clinical units, hospitals, supplier networks, and the institutional field. In the following section, we build on this critique to articulate theoretical insights that health care management research can offer to advance understanding of environmental sustainability, both within health care and in management theory more broadly.

## Insight and Theoretical Advance

The preceding analysis demonstrates that the difficulty of advancing environmental sustainability in health care does not stem primarily from sectoral resistance or implementation failure. Rather, it reflects theoretical underdevelopment in how management research conceptualizes (1) distant-future pursuit, (2) leadership in extra-professional domains, and (3) decision-making under asymmetry. Health care makes these weaknesses particularly visible, but they are not unique to the sector. In this section, we articulate how health care management research can advance by reframing environmental sustainability not as an ancillary concern, but as a distinct mode of managerial and organizational work. We advance three theoretical insights and summarize our analysis and suggestions for future work in Table 1.

**TABLE 1:** Framework for health care management research to advance environmentally sustainable health care

Core issue	Limitation in dominant theories	Theoretical insight/advance	Priority research directions	Implications for practice
Pursuing distant-future goals	Regard the future as a strategic backdrop or planning horizon rather than an object of sustained organizational work. Long-term orientation is assumed, not explained.	Shift from “long-term orientation” to active pursuit of distant futures as ongoing organizational work (futurism).	Longitudinal studies that collect rich data of how health-care organizations construct, sustain, and revise distant sustainability goals.	Actively sustain future-oriented goals over time (e.g., climate neutrality by 2050).
Leading sustainability beyond formal authority	Conceptualize leadership as hierarchical or professional Acknowledge but weakly theorize distributed leadership, especially outside core professional domains.	Understand distributed and extra-professional leadership, where authority and expertise are fragmented and sustainability lacks clear professional ownership.	Study boundary-spanning and orchestration practices in sustainability initiatives.	Lead sustainability initiatives without relying solely on formal authority.
Decision-making under asymmetry	Assume alignment between decision-makers and beneficiaries Value creation and capture are central Weakly theorize harm reduction and externalized benefits.	Reframe sustainability as governing asymmetric costs and benefits and managing environmental harm alongside quality, safety, and equity.	Study how organizations justify mitigation decisions with diffuse, delayed benefits. Study how to integrate temporal and geometric asymmetry in performance assessment.	Develop decision and performance frameworks capable of integrating temporally and spatially uneven payoffs.

### From Long-term Orientation to Active Pursuit of Distant Futures

A first theoretical advance concerns how management theory conceptualizes time and future-oriented action. Existing sustainability research often relies on notions such as long-term orientation, strategic foresight, or future commitment to explain organizational engagement with environmental goals. While these concepts acknowledge temporal extension, they tend to treat distant futures as relatively stable reference points rather than as outcomes of sustained organizational work.

Building on research on organizational futurity, health care management research can advance theory by shifting attention from whether organizations possess distant-future sustainability goals to how they actively pursue distant futures over time. This perspective foregrounds the ongoing practices through which distant environmental goals, such as climate neutrality or net-zero supply chains, are constructed, maintained, translated, and sometimes weakened in everyday organizational life.

Crucially, advancing this line of inquiry requires not only new concepts but also methodological approaches capable of capturing distant-future pursuit. Much existing empirical work on sustainability relies on cross-sectional designs, retrospective accounts, or performance correlations. These approaches are poorly suited to examining how distant futures shape or can inform present decision-making, particularly when outcomes unfold over decades.

Here, futurism and scenario building represent underutilized but promising methodological resources for health care management research. As developed in organization theory, futurism treats the future not as a forecast but as an object of organizational construction, enabling scholars to study how actors imagine, narrate, and act toward multiple possible futures. Scenario building, in turn, allows for examining how organizations explore alternative future pathways, assess trade-offs, and rehearse long-term consequences of present decisions.

Applied to environmentally sustainable health care, these methods can support several lines of inquiry that remain largely unexplored, including how health care organizations (can) use scenarios to connect distant-future environmental goals with near-term clinical, operational, and investment decisions; how competing sustainability futures (e.g., incremental efficiency versus transformative mitigation) are negotiated among managers, clinicians, and external stakeholders; how scenario-based engagement influences organizational learning, prioritization, and persistence in mitigation efforts over time.

Importantly, futurism and scenario building do not presume that organizations can predict or control the future. Rather, they make visible the assumptions, power relations, and temporal structures that shape which futures are taken seriously and which are marginalized. In health care, where environmental sustainability competes with immediate clinical demands and short planning cycles, these methods offer a way to study how distant futures gain or fail to gain traction in decision-making.

By incorporating futurism and scenario building into sustainability research, healthcare management scholars can move beyond static notions of long-term orientation and develop empirically grounded theories of how organizations pursue distant environmental futures and with what consequences. Over time, the health impacts of climate and environmental change have become increasingly well understood, making longer-term perspectives more relevant to the present-day scope of health care managers’ roles.

### Leadership Beyond Authority and Profession: Theorizing Extra-professional Sustainability Work

A second theoretical advance concerns leadership and the actions leaders take. Our theoretical analysis showed that environmental sustainability in health care is not only distributed across organizational boundaries, but also extra-professional: it lies outside the established jurisdictions of both managerial and clinical professions.

Health care management research can advance management theory by explicitly theorizing extra-professional leadership as not only individual action, but also collective action oriented toward issues that lack clear professional ownership yet require sustained coordination across actors with different forms of expertise and authority.

This advance involves shifting from leader-centric or authority-based models toward an understanding of leadership as boundary-spanning work that creates legitimacy for new concerns; orchestration among actors who do not share hierarchical relationships; translation between professional logics, managerial priorities, and external societal expectations.

Environmental sustainability makes the need for such theorizing especially salient because it cannot be fully absorbed into existing professional scripts without transformation. Health care thus exposes a general limitation in management theory: its difficulty accounting for leadership when neither hierarchical authority nor professional expertise provides a stable foundation for action.

By studying how health care managers and clinicians collectively engage with sustainability as an extra-professional concern, health care management research can contribute new theoretical insight into how novel issues become integrated into organizational and professional practice.

### From Value Alignment to Governing Asymmetric Costs and Benefits

A third theoretical advance concerns decision-making rationality. Dominant management theories implicitly assume that organizational action is sustained when costs and benefits can be aligned, through incentives, value capture, or stakeholder pressure. Environmental mitigation in health care systematically violates this assumption.

Health care management research can advance theory by developing a more explicit account of how organizations govern decisions under conditions of asymmetric costs and benefits, where costs are immediate, localized, and visible; benefits are diffuse, delayed, and often external to the organization; many beneficiaries lack representation in decision-making processes. Traditionally, health care outcomes have been assessed at the individual or population level, focusing primarily on the immediate and direct implications of care for the recipient. This approach has been instrumental in improving health and quality of life within defined contexts. The integration of sustainable health care practices requires expanding the scope of outcome assessment to consider the long-term and widespread implications of health care practices. This includes evaluating how actions taken today may impact the health and well-being of future generations and how (un)sustainable practices in one region can exacerbate or improve health outcomes elsewhere, particularly in areas already disproportionately affected by climate change (Bianco et al., 2024).

Rather than treating such asymmetry as a barrier to be overcome, this perspective treats it as a constitutive feature of sustainability management. Health care is a revealing context because harm prevention is already central to its ethical and professional foundations, yet environmental harm remains weakly integrated into managerial decision frameworks.

Health care management research can contribute by examining how environmental considerations are weighed alongside quality, safety, and equity, and how organizations justify mitigation efforts that do not produce immediate organizational returns. Expanding this assessment framework provides an opportunity for health care management research to develop tools and methodologies for measuring these wider impacts, such as intergenerational health effects and global equity. For example, Grant et al. (2007) show that making the beneficiaries of professional or organizational work visible to those performing the work can strengthen motivational persistence. In their study of a fundraising organization, exposure to beneficiaries increased fundraisers’ persistence and performance. Albeit in a different empirical context, these findings suggest a relevant underlying mechanism: increasing the salience of future beneficiaries may motivate health care managers to engage more persistently in sustainable healthcare practices. Thus, exploring how current and future beneficiaries of sustainable health care practices can be made more salient to current healthcare managers may offer a promising pathway.

## Practical implications

The theoretical advances outlined above have important implications for management practice in health care. These implications are not prescriptive “best practices,” since this would be premature, but clarifications of why existing managerial tools often fall short and where new forms of practice are required.

### Managing Sustainability as Ongoing Future-oriented Work

First, health care managers should recognize that articulating distant-future sustainability goals is only a starting point. The primary managerial challenge lies in sustaining the relevance of these futures over time. This implies attending to the temporal infrastructures of organizations, including governance, planning cycles, investment horizons, and performance metrics, and considering how they enable or undermine distant-future environmental goals.

Practically, this may involve creating intermediate targets, narrative practices, and review processes that repeatedly reconnect present decisions to distant environmental objectives, rather than relying on one-time commitments or vision statements (Feuls et al., 2025).

### Leading Sustainability Without Relying on Authority or Profession

Second, health care managers should anticipate that environmental sustainability will not fit neatly within existing lines of authority or professional responsibility. Effective leadership in this domain requires convening and coordinating actors across professional and organizational boundaries, often without formal control.

This suggests a shift in managerial focus from directing action to enabling collaboration, building shared understanding, and legitimizing environmental considerations as relevant to core clinical and organizational concerns. Managers who approach sustainability primarily as a compliance or efficiency issue are likely to encounter resistance or superficial adoption.

### Making Decisions Under Asymmetry Explicit Rather Than Implicit

Third, health care managers should expect environmental mitigation decisions to involve uneven distributions of costs and benefits. Rather than attempting to mask or fully align these asymmetries, managers may be better served by making them explicit and integrating environmental harm into deliberative decision processes alongside quality, safety, and equity. Doing so does not eliminate trade-offs, but it clarifies the basis on which decisions are made and reduces the risk that environmental considerations are consistently deferred because they lack immediate organizational payoff.

Finally, while we have highlighted fundamental conceptual questions that require careful scholarly attention to support managers, this need for deeper theorizing should not be read as a call to delay action. Precisely because research on sustainable health care practices remains nascent and evolving, collaboration between scholars and practitioners is also essential now. Advancing environmentally sustainable health systems will depend on the same two-way translation that has long characterized health care management research: designing and studying concrete interventions while learning from ongoing practice to refine theory. Generating evidence, experimenting with sustainable practices, and developing new measurement and governance instruments must therefore occur in parallel, and many of these initiatives can be addressed through existing frameworks that health care management scholars have offered. Clarifying the phenomenon is meant to strengthen, not postpone, the efforts of managers tasked with reducing harm to both present and future patients worldwide.
